# Breast cancers with high DSS1 expression that potentially maintains BRCA2 stability have poor prognosis in the relapse-free survival

**DOI:** 10.1186/1471-2407-13-562

**Published:** 2013-12-01

**Authors:** Andri Rezano, Kazuhiko Kuwahara, Mutsuko Yamamoto-Ibusuki, Masahiro Kitabatake, Penpak Moolthiya, Suchada Phimsen, Taiji Suda, Shigenobu Tone, Yutaka Yamamoto, Hirotaka Iwase, Nobuo Sakaguchi

**Affiliations:** 1 Department of Immunology, Graduate School of Medical Sciences, Kumamoto University, 1-1-1, Honjo, Chuo-ku, Kumamoto 860-8556, Japan; 2 Department of Breast and Endocrine Surgery, Graduate School of Medical Sciences, Kumamoto University, 1-1-1, Honjo, Chuo-ku, Kumamoto 860-8556, Japan; 3 Department of Biochemistry and Electron Microscope Center, Kawasaki Medical School, 577 Matsushima, Kurashiki, Okayama 701-0192, Japan

**Keywords:** Breast cancer, BRCA2, Cancer prognosis, DNA damage, DSS1

## Abstract

**Background:**

Genetic BRCA2 insufficiency is associated with breast cancer development; however, in sporadic breast cancer cases, high BRCA2 expression is paradoxically correlated with poor prognosis. Because DSS1, a mammalian component of the transcription/RNA export complex, is known to stabilize BRCA2, we investigated how the expression of DSS1 is associated with clinical parameters in breast cancers.

**Methods:**

*DSS1* mRNA and p53 protein were examined by RT-PCR and immunohistochemical staining of breast cancer specimens to classify *DSS1*^high^ and *DSS1*^low^ or p53^high^ and p53^low^ groups. Patient survival was compared using Kaplan-Meier method. *DSS1*^high^ or *DSS1*^low^ breast cancer cells were prepared by retroviral cDNA transfection or *DSS1* siRNA on proliferation, cell cycle progression, and survival by flow cytometric analyses with or without anti-cancer drugs.

**Results:**

In comparison to patients with low levels of DSS1, high-DSS1 patients showed a poorer prognosis, with respect to relapse-free survival period. The effect of DSS1 was examined in breast cancer cells *in vitro*. DSS1 high-expression reduces the susceptibility of MCF7 cells to DNA-damaging drugs, as observed in cell cycle and apoptosis analyses. *DSS1* knockdown, however, increased the susceptibility to the DNA-damaging drugs camptothecin and etoposide and caused early apoptosis in p53 wild type MCF7 and p53-insufficient MDA-MB-231 cells. *DSS1* knockdown suppresses the proliferation of drug-resistant MDA-MB-231 breast cancer cells, particularly effectively in combination with DNA-damaging agents.

**Conclusion:**

Breast cancers with high DSS1 expression have worse prognosis and shorter relapse-free survival times. DSS1 is necessary to rescue cells from DNA damage, but high DSS1 expression increases drug resistance. We suggest that DSS1 expression could be a useful marker for drug resistance in breast cancers, and DSS1 knockdown can induce tumor apoptosis when used in combination with DNA-damaging drugs.

## Background

Breast cancer development is associated with various molecular abnormalities in genes involved in DNA repair, cell cycle control, signal transduction, and tumor suppressor function; these are the predisposing hereditary causes in approximately 5-10% of breast cancers [1]. Hereditary breast cancers exhibit germline mutations of BRCA1 and BRCA2 at certain incidences. Most breast cancer cases with germline BRCA2 mutations have loss of heterozygosity at the *BRCA2* locus, resulting in the loss of the *BRCA2* allele [2,3]. BRCA2 deficiency is associated with various abnormalities in the response to DNA cross-linking agents, such as defects in homologous recombination (HR), formation of RAD51 foci, DNA replication, and checkpoint regulation [4-9].

In contrast, in the majority (90%) of sporadic breast cancers, BRCA2 is not mutated [10]. Rather, the expression of BRCA2 is increased in tumors, as shown in reverse transcription (RT)-PCR, quantitative RT-PCR (qRT-PCR), and immunohistochemical analyses [11]. BRCA2 is significantly over-expressed in sporadic breast, ovarian, pancreatic, and prostatic cancers [12]. BRCA2 over-expression, but not decreased expression, was correlated with histopathological grade III; this over-expression, which is attributable to nuclear polymorphism, was also correlated with the mitotic index, implicating a close association between BRCA2 over-expression and the proliferation rate of breast cancer cells [11,13]. Furthermore, a three-gene expression signature (*BRCA2, DNMTB3,* and *CCNEI*) was found to be an independent prognostic marker in breast cancer [14]. A high BRCA2 level is associated with poor outcome and correlated with high proliferation rate. In a hierarchical clustering analysis of 47 candidate genes, BRCA2 was found to be the leading gene in a cluster of proliferation-associated genes. The finding was supported by an *in vitro* study in which BRCA2 over-expression suppressed HR and reduced RAD51 foci formation, along with inactivation of p53, which suggests that moderate levels of BRCA2 play a role in the stimulation of HR for appropriate DNA repair [15].

The expression level of BRCA2 is presumably regulated through various mechanisms including transcription, subcellular localization, binding to partners, and protein modification and stabilization. A stabilization factor of BRCA2, deleted in split hand/split foot 1 (DSS1), was originally identified as a BRCA2-associated protein, and its depletion was shown to induce BRCA2 destabilization [16]. DSS1 is a candidate gene for an inherited limb development disease and is located on chromosome 7q21.3–q22.1. DSS1 is a principal component of the mammalian mRNA transcription/exportation 2 (TREX2) complex that includes GANP, PCID2, and DSS1 and interacts with various components of RNA metabolism including RNA polymerase II, RNA splicing factors, and helicases [17]. *Saccharomyces* deficient in the components of the TREX2 complex displayed abnormalities in cell proliferation and cell cycle control, but abnormal expression of individual components of TREX2 results in different phenotypes in mammalian cells. For example, mammalian GANP insufficiency causes DNA injuries during proliferation and is associated with tumor development in human glioblastoma [18]. Loss of PCID, another TREX2 component, causes a severe defect in Mad2 expression with a marked reduction in *Mad2* mRNA export, which causes severe hyperploidy and apoptotic cell death [19]. However, increased expression of TREX2 components, in contrast to reduced expression, has rarely been shown to be associated with tumor development.

Given that the BRCA2-expression is correlated with poor prognosis in clinical cases [11,13], we investigated the outcome of abnormal DSS1 expression in human breast cancer cases. DSS1 is certainly expressed at high levels in a group of breast cancer cases with poor prognosis. The imbalance of DSS1 over-expression associated with BRCA2 expression could affect breast cancer development. Here, we demonstrate that increased DSS1 expression is correlated with chemo-resistance in sporadic breast cancers, which might be responsible for the worse prognosis of patients with high *DSS1* levels, particularly with respect to relapse-free survival (RFS).

## Methods

### Patients and breast cancer tissues

Breast tumor specimens from 289 female patients with invasive breast carcinoma, who were treated at Kumamoto University Hospital between 2001 and 2009, were included in this study. Among these patients, p53 immunohistochemical data were available for 227 (78.5%) patients. The patients were from a consecutive series; those with other malignancies or bilateral breast cancer were excluded. Samples were snap frozen in liquid nitrogen at the time of the pretherapeutic biopsy or surgical treatment and stored at -80°C until simultaneous total RNA extraction. The median age of the patients was 59 years (range, 21–93 years). Adjuvant treatment and neoadjuvant treatment were decided by risk evaluation according to tumor biology [estrogen receptor alpha (ERα), progesterone receptor (PgR), and HER2 but not Ki-67 status] and clinical staging, including sentinel lymph node biopsy, in accordance with the recommendations of the St. Gallen international expert consensus on the primary therapy of early breast cancer. In detail, neoadjuvant treatments were administered to 62 patients, 46 of whom received chemotherapy and 16 of whom received hormonal therapy. The breast-conserving rate was 68.2%, and most of these were treated with radiotherapy. Axillary lymph node dissection was carried out in 45.2% of cases; the others were spared dissection due to negative lymph node status by sentinel node exploration. A total of 208 patients were treated with hormone therapy, 106 patients were given chemotherapy, and 19 patients were treated with trastuzumab. The ethics committee of Graduate School of Medical Sciences, Kumamoto University approved the study protocol. Informed consent was obtained from all patients. Patients received follow-up studies every 3 months. The median follow-up period was 66 months (range 15–144 months).

### RNA extraction, primers, and qRT-PCR

Total RNA was isolated from tissue specimens using the Allprep DNA/RNA Mini Kit (Qiagen, Germantown, MD). Total RNA (0.5 μg) was reverse transcribed to cDNA using PrimeScript RT reagent Kit (Takara Bio Inc., Otsu, Japan), according to the manufacturer’s protocol. Each PCR was performed using 2 μl of cDNA and 0.2 μmol/l of each probe in a LightCycler System with SYBR Premix Dimer Eraser (Takara Bio Inc.). PCR primer sequences were as follows: for *DSS1*, forward 5′-GTTAGAGGAAGACGACGAGT-3′ and reverse 5′-GGATGCTATGAAGTCTCCAT-3′; for *β-actin*, forward 5′-TGGCACCCAGCACAATGAA-3′ and reverse 5′-CTAAGTCATAGTCCGCCTAGAAGCA-3′. Each reaction (20 μl samples) was performed under the following conditions: initialization for 10 sec at 95°C, and then 45 cycles of amplification, with 5 sec at 95°C for denaturation and 20 sec at 60°C for annealing and elongation. The expression level of *DSS1* mRNA is given as relative copy numbers normalized to those of *β-actin* mRNA. In some experiments, qRT-PCR was performed using a LightCycler (Roche Diagnostics, Indianapolis, IN). Specific oligonucleotide primers and probes for *DSS1* and *gapdh* were purchased (Nihon Gene Research Laboratories, Sendai, Japan). The specific primers for *DSS1* were the same as above. Their sequences were as follows: *dss1 donor* probe, 5′-CCCAATTATCCTCCCAGACATGTGCATCTT-3′; *dss1 acceptor* probe, 5′-ATCTTCATCTAAGCCAGCCCAGTCTTCGG-3′; *gapdh-*F, 5-CAGCCTCAAGATCATCAGC-3′; *gapdh*-R, 5′-GGCCATCCACAGTCTTCT-3′; *gapdh donor* probe, 5′-GGTCATCCATGACAACTTTGGTATCGTGGAA-3′; *gapdh acceptor* probe, 5′-GACTCATGACCACAGTCCATGCCATCACTG-3′. The level of *DSS1* mRNA expression was determined relative to *gapdh*.

### Immunohistochemistry and scoring system

Histological sections (4 μm) were deparaffinized and incubated for 10 min in methanol containing 0.3% hydrogen peroxide. Mouse monoclonal antibodies (mAbs) against ERα (SP1, Ventana Japan, Tokyo, Japan), PgR (1E2, Ventana Japan) and Ki67 (MIB1, Dako Japan, Tokyo, Japan), a polyclonal Ab against HER2 (Dako Japan), and a mouse mAb against p53 (DO7, Dako Japan) were used; staining was carried out in the NexES IHC Immunostainer (Ventana Medical Systems, Tucson, AZ), in accordance with the manufacturer's instructions. ERα and PgR status were evaluated based on the percentage of positively stained nuclei, and the status of a nucleus was considered positive when ≥1% of the nucleus was stained. HER2 was evaluated using the HercepTest method (Dako Japan), with membranous staining scored on a scale of 0 to 3+. Tumors with scores of ≥3 or with a ≥2.2-fold increase in *HER2* gene amplification as determined by fluorescence *in situ* hybridization were considered to be positive for HER2 over-expression. Ki67 was scored as the percentage of nuclear-stained cells out of all cancer cells along the invasive front of the tumor in × 400 high-power fields; this gave the Ki67 labeling index. p53 was evaluated based on the percentage of positively stained nuclei, and the status of a nucleus was considered positive when ≥20% of the nucleus was stained.

### Cell lines and small interfering RNA (siRNA) treatment

The human breast cancer cell lines MCF7 and MDA-MB-231 were obtained from the American Type Culture Collection and maintained in RPMI-1640 (Life Technologies, Carlsbad, CA) supplemented with 10% heat-inactivated fetal calf serum (MP Biomedicals, Santa Ana, CA), 2 mM L-glutamine (Lonza, Allendale, NJ), and 5 × 10^-5^ M 2-mercaptoethanol (Wako Pure Chemicals Industries, Osaka, Japan) with 5% CO_2_ at 37°C. siRNA treatment was performed when the cells reached 50% confluence; cells were transfected with 10 nM of control (siCtrl) or *DSS1* (si*DSS1*; Life Technologies) siRNA using Lipofectamine RNAiMAX (Life Technologies). The siRNA target sequences were as follows: si*DSS1-*(a), 5′-GACAAUGUAGAGGAUGACUUCUCUA-3′; si*DSS1-*(b), 5′-GCAGCCGGUAGACUUAGGUAUGUUA-3′; siCtrl-(a), 5′-GACGUAUAGGGUAAGUCCUUAACUA-3′; siCtrl-(b), 5′-GCAGGCGAUUCAGAUCUGGUGCUUA-3′. The results with si*DSS1*-(a) are shown in the figures as representatives.

### Establishment of DSS1 over-expressing cells

A retroviral vector, designated pFB-DSS1-IRES-GFP, was transfected into PLAT-GP (Cellbiolabs, San Diego, CA) retrovirus packaging cells using FuGENE HD (Roche Diagnostics). MCF7 and MDA-MB-231 cells were infected with the retroviruses using polybrene (8 μg/ml; Sigma, St. Louis, MO) for 2 days. GFP-positive cells were sorted using a JSAN cell sorter (BayBioscience, Kobe, Japan).

### Cell proliferation assay

*In vitro* cell proliferation was determined using an MTT assay performed with a Cell Proliferation Kit I (Roche Applied Science, Penzberg, Germany). Briefly, at 24 hr after siRNA treatment, 2.5 × 10^3^ cells per well were seeded and incubated with MTT labeling reagent (0.5 mg/ml) for 6 hr at 37°C. The soluble formazan product was quantified using an ELISA reader at 590 nm from day 1 to day 4.

### Detection of dead cells

DNA damage-inducing agents were added when the cells reached 80% confluence. CPT (Merck Millipore, Billerica, MA) or ETP (Merck Millipore) was introduced to the cells at a final concentration of 50 μM. After 24 to 72 hr, cells were harvested, washed with ice-cold PBS, resuspended in PBS with 250 μg/ml RNase A, and stained with 2× PI solution for 2 hr at 4°C. The cell cycle was analyzed using FACSCalibur (BD, Franklin Lakes, NJ) and CellQuest software.

### Annexin XII/PI staining

Cells were washed twice with staining buffer (0.1 M Hepes pH 7.4, 1.4 M NaCl, 25 mM CaCl_2_) and resuspended in FITC-conjugated Annexin XII (Abcam, Cambridge, UK) on ice for 10 min. Cells were then counterstained with 0.5 μg/ml PI on ice for 5 min, and an equal volume of staining buffer was added. Early apoptotic (Annexin XII^+^/PI^-^) and late apoptotic/necrotic (Annexin XII^+^/PI^+^) cells are shown with merged color.

### EM analysis

After *DSS1* knockdown, floating cells were centrifuged in microfuge tubes and then fixed in 2.5% glutaraldehyde in 0.1 M cacodylate buffer (pH 7.2) containing 0.1% CaCl_2_. After washing in the same buffer, embedding in 2% agarose and incubating post-fixation in 1% OsO_4_ for 1 hr, the specimens were washed again, dehydrated in a series of ethanol baths, and embedded in Epon 812. Thin sections were cut with a Leica Ultracut UCT microtome, post-stained with 2% uranyl acetate and Reynolds lead citrate, and examined using a JEOL JEM 1400 operated at 80 kV. Two researchers (TS and ST) independently analyzed cells with apoptotic changes using digitized EM images.

### Alkaline comet assay

DNA damage induced by DSBs and single-strand breaks were analyzed using a comet assay kit (Trevigen, Inc., Gaithersburg, MD), according to the manufacturer’s protocol. For quantification, the tail moment (an index of DNA damage calculated as the product of the tail length and the fraction of DNA in the comet tail) was evaluated using CometScore version 1.5 software (AutoComet.com). At least a hundred cells from each sample were scored.

### Statistical analysis

The nonparametric Wilcoxon (for uni-variable), Kruskal-Wallis test (for multi-variables), and the χ^2^ test were adopted for statistical analysis of the associations between the *DSS1* mRNA status and various clinicopathological factors. RFS and BCSS curves were calculated according to the Kaplan-Meier method and verified by the log-rank test. All statistical significance was defined as *P* < 0.05. JMP software version 8.0.2 for Windows (SAS institute Japan, Tokyo, Japan) was used for all statistical analyses.

## Results

### Increased DSS1 expression in breast cancer cases with poor prognosis

**Figure 1:**
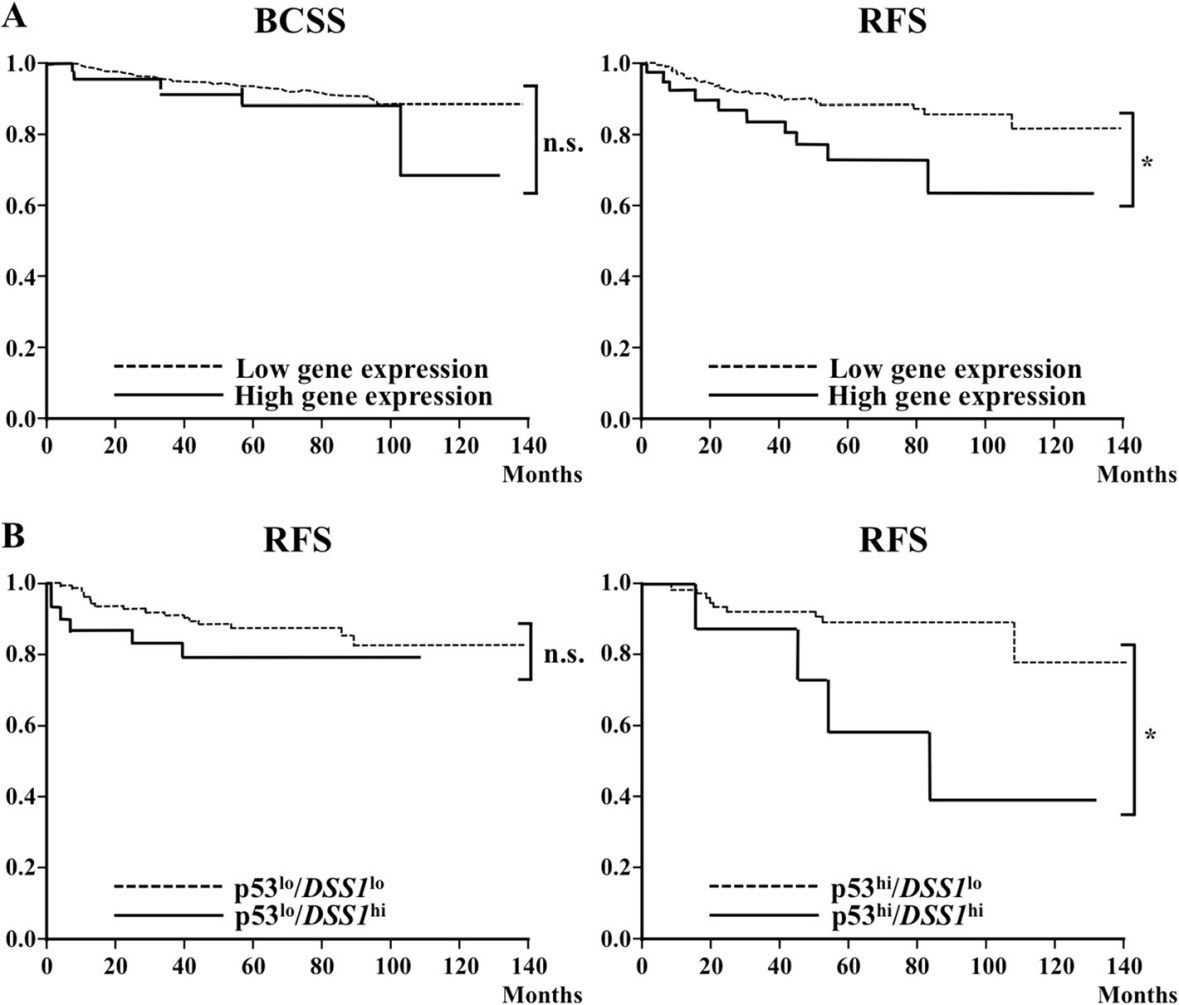
**Survival of patients with primary breast cancers.** Patient survival was assessed using the Kaplan-Meier method. Clinical breast cancer patients were followed for more than 120 months and classified into groups based on the expression of *DSS1* mRNA, which was determined using qRT-PCR of total RNAs from 289 primary invasive breast cancer patients. **(A)** BCSS (left) and RFS (right) of the DSS1 low (n = 248) and high (n = 41) groups are shown. Statistical significance was calculated as log-rank: ^n.s.^*P* = 0.25 (left) and *P = 0.011 (right), respectively. **(B)** The patient survival was compared using double markers with p53 and *DSS1* mRNAs as p53^low^/*DSS1*^low^ (n = 120), p53^low^/*DSS1*^high^ (n = 31), p53^high^/*DSS1*^low^ (n = 85), and p53^high^/*DSS1*^high^ (n = 10) groups. Statistical significance was calculated as log-rank: ^n.s.^*P* = 0.49 (left) and *P = 0.045 (right), respectively.

We examined the survival of breast cancer patients using the Kaplan-Meier method. We analyzed the expression level of *DSS1* mRNA by qRT-PCR from the tumor samples obtained at surgery using primers for *DSS1* mRNA (Additional file 1: Figure S1). The relative expression level was arbitrarily determined and used for classification of breast cancer cases into *DSS1*-high (*DSS1*^high^) and DSS1-low (*DSS1*^low^) groups. The *DSS1*^high^ group showed a significant shorter survival time (*P = 0.011) on the RFS curve, while the difference in the breast cancer specific survival (BCSS) curves was not significant (n.s.; *P* = 0.25) (Figure 1A). DSS1 physically interacts with the RPN3/S3 proteasomal subunit, which increases the degradation of ubiquitinated p53 [20]. In a large cohort, over-expression of p53 was significantly associated with poor prognosis in premenopausal women treated with tamoxifen after chemotherapy [21]. The p53 expression was used for classification by arbitrarily determined conditions (see Methods). We compared RFS among p53^low^/*DSS1*^low^, p53^high^/*DSS1*^low^, p53^low^/*DSS1*^high^, and p53^high^/*DSS1*^high^ groups (Figure 1B). While the number of cases was small, no change of the survival curve was observed when p53 expression was low; however, the *DSS1*^high^ group showed a worse prognosis in comparison with the *DSS1*^low^ group in breast cancer cases with high p53 expression (*P = 0.045). Other parameters and markers were compared in the *DSS1*^high^ and *DSS1*^low^ groups (Table 1). No significant differences were observed in the groups of pre- or post-menopausal women, positive or negative nodal status, tumor size < 20 or ≥ 20 mm, nuclear grade I to III, positive or negative vessel invasion score, ERα status, and PgR status. However, *DSS1* expression showed a significant difference (*P = 0.0255) based on HER2 status, which is presumably associated with effective treatment using trastuzumab as the standard regimen for HER2-positive breast cancer cases. Interestingly, *DSS1* expression was markedly increased (55.6; median of *DSS1* relative expression) in the group of breast cancer cases with low Ki67 labeling indices (< 20%) compared with the expression (36.0; median of *DSS1* relative expression) in the group with high Ki67 labeling indices (≥ 20%).

**Table 1:** **Comparison of *DSS1 *** **gene expression among various groups of breast cancer patients**

**Clinical parameters**	**Group**	**Number of patient**	***DSS1 *** **gene expression median (25%, 75%)**	**Mann–Whitney U-test (*P*)**
Menopause	Pre-	78	48.7 (8.05, 143)	0.554
	Post-	210	49.2 (8.09, 92.4)	
Nodal	(-)	179	41.2 (7.04, 94.2)	0.132
status	(+)	110	64.9 (13.2, 117)	
Tumor	< 20	125	44.3 (8.08, 108)	0.646
size (mm)	≥ 20	164	52.2 (8.41, 104)	
Nuclear	1	143	51.7 (8.33, 114)	0.703
grade	2	74	39.7 (8.24, 90.0)	
	3	72	57.5 (6.19, 91.3)	
Vessel	(-)	187	40.0 (5.47, 88.9)	0.308
invasion	(+)	77	47.1 (11.5, 107)	
ERα	(-)	65	52.7 (5.71, 105)	0.962
	(+)	224	49.0 (8.36, 102)	
PgR	(-)	96	60.3 (6.14, 103)	0.487
	(+)	193	47.1 (8.37, 106)	
HER2	(-)	248	53.2 (10.6, 115)	*0.0255
	(+)	41	21.8 (3.78, 74.8)	
Ki67	< 20	113	55.6 (16.4, 132)	**0.0096
(%)	≥ 20	169	36.0 (4.97, 82.0)	

(*) and (**) denote *P* < 0.05 and *P* < 0.01, respectively.

The data suggest that the high *DSS1* expression may not be directly associated with the proliferation of breast cancer cells in humans.

### Effect of increased DSS1 expression on resistance to chemotherapy

**Figure 2:**
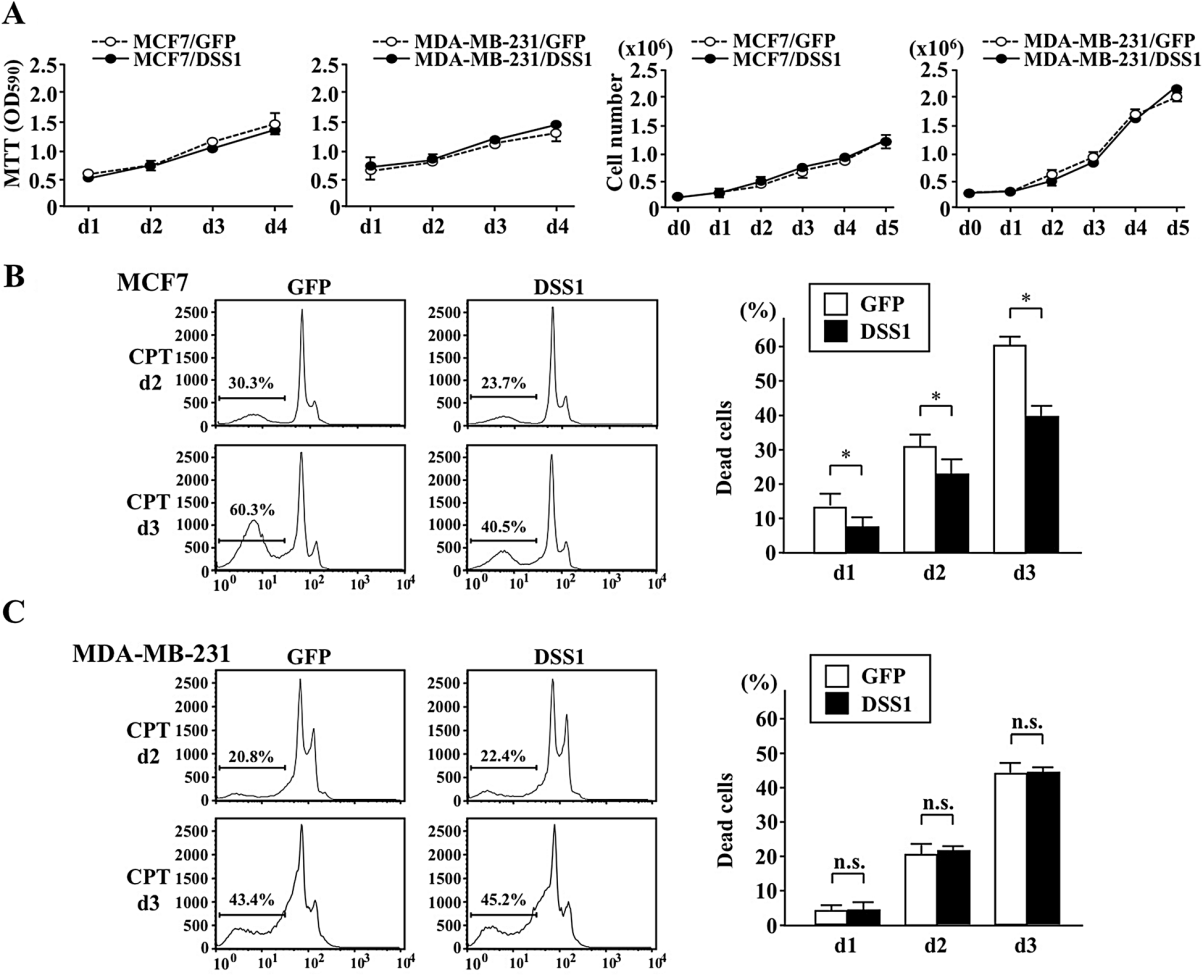
**Effect of DSS1 over-expression on proliferation of the breast cancer cell lines MCF7 and MDA-MB-231.** Human *DSS1* cDNA was introduced using an ecotropic retroviral vector with *GFP* plus *IRES* sequence. **(A)***DSS1* + *GFP* vector and *GFP* alone vector transfectants were sorted and used for cell proliferation assay on day 4 to day 5 after transfection. *DSS1* + *GFP* and *GFP* alone transfectants showed similar proliferation rates in the MTT assay (left) and cell counting (right). **(B)** Drug sensitivity was examined in the MCF7 transfectants using an optimized dose of CPT (50 μM) for 2 to 3 days. Results of the cell cycle profile (left) are shown in the graph, which represents more than three independent experiments. The proportions of dead cells were measured by counting the sub-G1 fraction and compared in the graph (right). Statistical significance was calculated using Student’s t-test, with *P < 0.05 as significant. **(C)** Drug sensitivity was similarly compared in MDA-MB-231 cells. n.s.: not significant.

**Figure 3:**
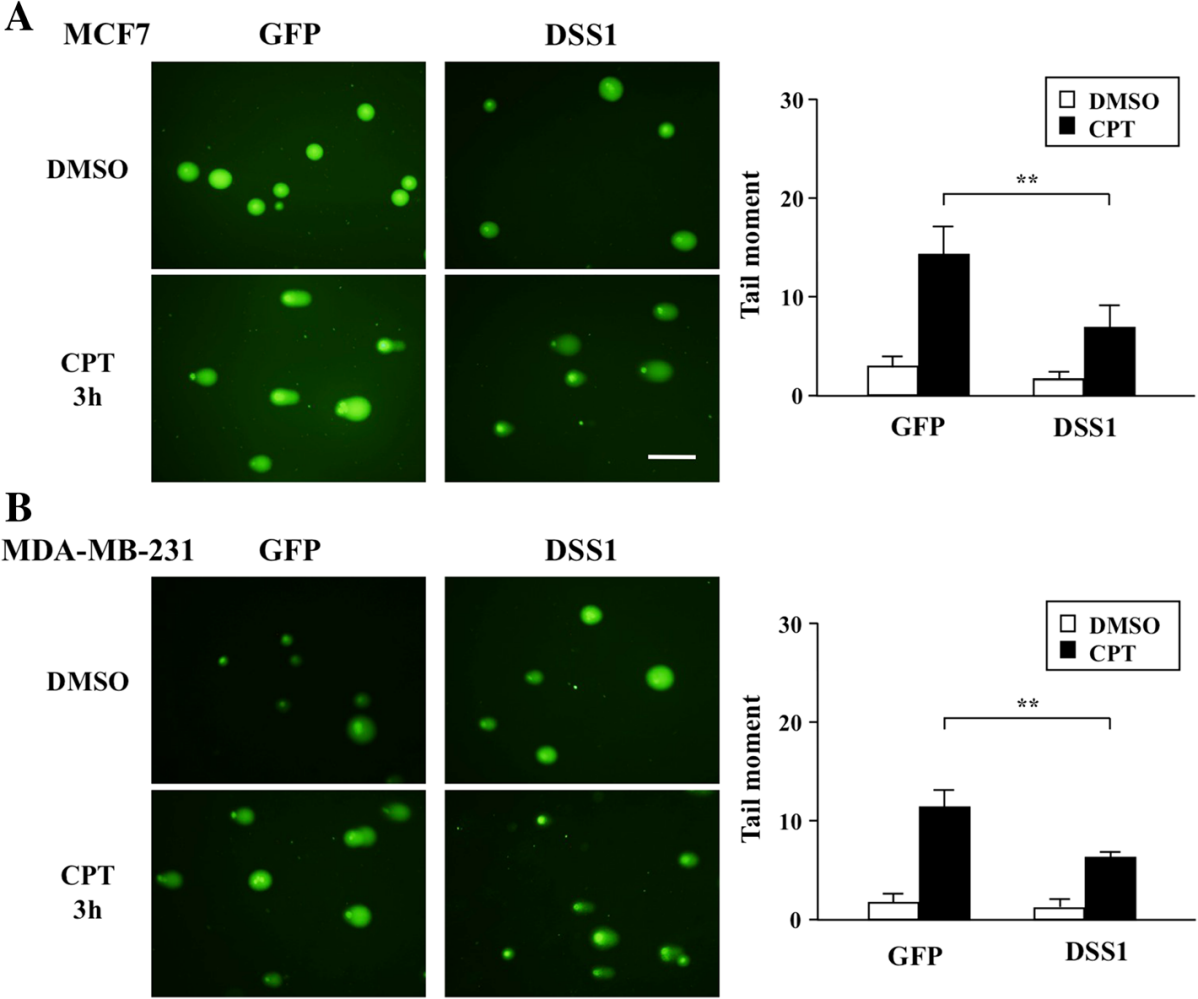
**Effect of DSS1 over-expression on sensitivity to DNA damage.** Effect of DSS1 over-expression on drug-induced DNA injury was measured using the alkaline comet assay after treatment of cells with CPT (50 μM) for 3 hr. DMSO was used as a solvent control. The DNA content pattern observed under fluorescent microscopy is shown (left), and the DNA damage rate was measured as the tail moment in the fluorescence image using CometScore software. DSS-over-expressing MCF7 **(A)** or MDA-MB-231 **(B)** cells were compared with GFP-expressing controls. Data obtained by counting more than 100 cells are shown as a graph, which represents more than three independent experiments (right). Statistical significance was calculated using Student’s t-test, with **P < 0.01 as significant. Scale bar indicates 100 μm.

For molecular analysis, we used two breast cancer cell lines, MCF7 (ERα^+^PgR^+^ with wild type p53) and MDA-MB-231 (ERα^-^PgR^-^ with p53 mutation). The effect of DSS1 over-expression on cell proliferation and cell cycle progression was examined in breast cancer cells that had been engineered to over-express DSS1 (MCF7^DSS1^ or MDA-MB-231^DSS1^) by retroviral transfection (Additional file 2: Figure S2). Stable transfectants that expressed high levels of *DSS1* mRNA did not show any changes in cell cycle or cell proliferation compared with the control (GFP only) transfectants (Figure 2A). DSS1-over-expressing cells showed no abnormalities in proliferation. Trastuzumab is a first choice for treatment of HER2-positive breast cancer in our cohort, but camptothecin (CPT) is often chosen as a regular regimen for HER2-negative breast cancer cases (Table 1). The susceptibility of DSS1-over-expressing breast cancer cells to the DNA-damage inducing agents CPT and etoposide (ETP) was examined. DSS1 over-expression renders breast cancer cells more resistant to treatment with CPT, an inhibitor of type 1 topoisomerase (which cleaves one strand of double-stranded DNA), compared with the GFP-control MCF7 transfectants (Figure 2B), while no change was detected in DSS1 over-expressing MDA-MB-231 transfectants (Figure 2C). The effect of DSS1 over-expression was masked in both MCF7 and MDA-MB-231 cells upon exposure to ETP, an inhibitor of topoisomerase II (which cleaves two strands of double-stranded DNA and induces cell cycle arrest at G2/M) (Additional file 3: Figure S3A and B). Therefore, DNA damage was examined using the alkaline comet assay (Figure 3A and B). DSS1 over-expression reduced CPT-induced DNA damage in MCF7 and MDA-MB-231 cells, suggesting that DSS1 over-expression increases drug resistance in breast cancers.

### Effect of DSS1 knockdown on the sensitivity of breast cancer cells carrying wild-type p53 or insufficient p53 to chemotherapeutics

**Figure 4:**
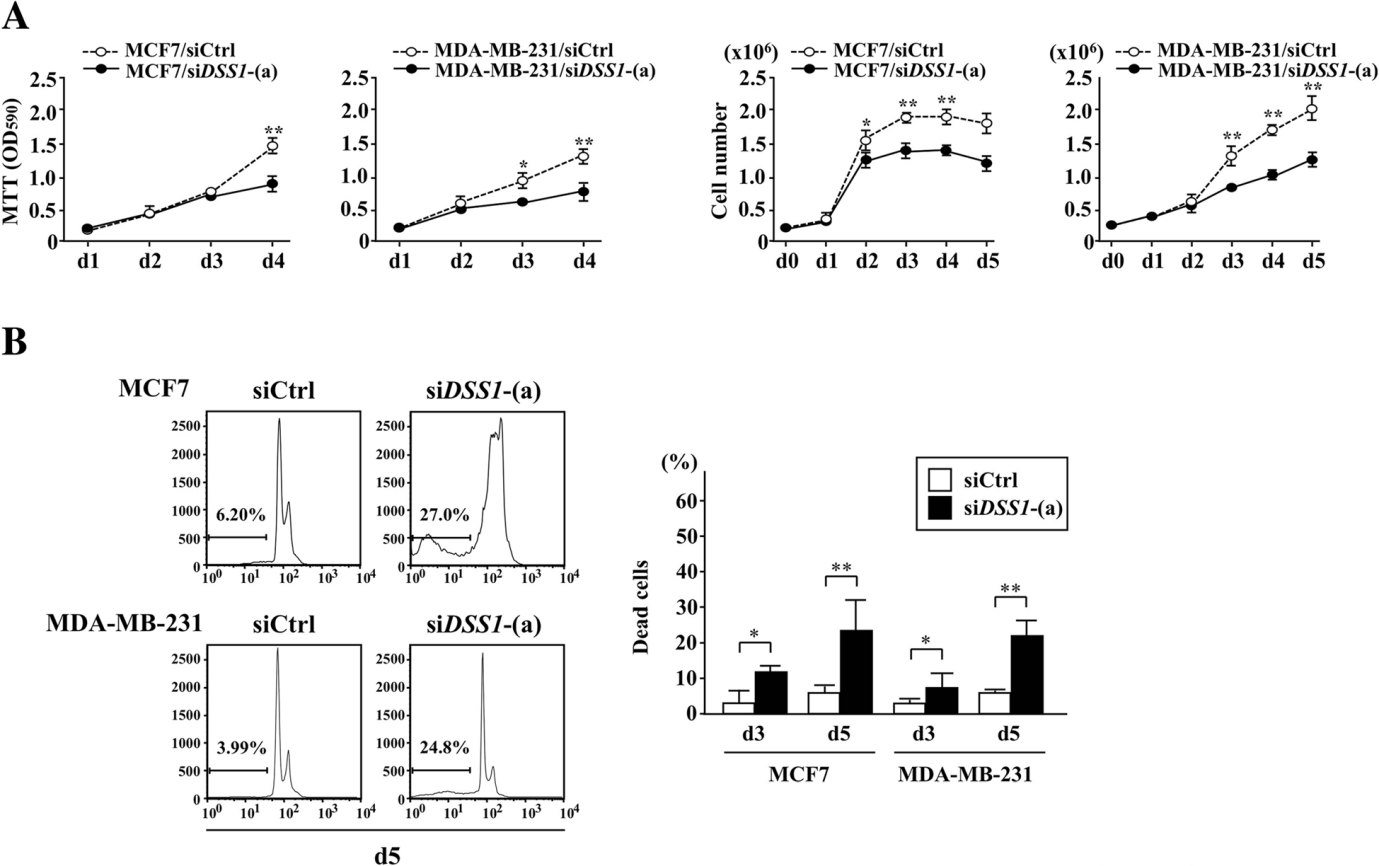
**Effect of *DSS1 *knockdown in the breast cancer cell lines MCF7 and MDA-MB-231.** *DSS1* knockdown was carried out in MCF7 and MDA-MB-231 cells by treatment with si*DSS1*. Two si*DSS1* sequences targeting two separate regions of the sequence showed similar knockdown efficiencies and similar effects on the proliferation of MCF7 and MDA-MB-231 cells (Figure S4). **(A)** The proliferation rates of siCtrl- and si*DSS1*-(a)-treated cells were assessed by MTT assay (left) and cell counting (right). **(B)** Cell cycle profiles of the transfectants were examined on day 3 and day 5 of culture (right; day 5 profile). The proportions of dead cells were measured by the counting sub-G1 fraction and are compared in the graph (right). The experiments were carried out more than three times, and the results are shown as an average. Statistical significance was assessed by Student’s t-test, with *P < 0.05 and **P < 0.01.

Next, we examined cell growth in *DSS1*-depleted cells. Two siRNAs directed against two independent *DSS1* mRNA sequences elicited marked reductions in *DSS1* mRNA levels (Additional file 4: Figure S4A) and protein levels (Additional files 4: Figure S4B and 5); we therefore used one of these sequences in the following experiments. *DSS1* knockdown (si*DSS1*) caused decreased cell growth, as observed in an MTT assay (Figure 4A: left) and in an assessment of cell number (right). At 3 and 5 days after si*DSS1*-treatment, the number of dead cells in the sub-G1 population increased compared to siCtrl-treated cells (Figure 4B). si*DSS1*-treated MCF7 cells experienced cell cycle arrest at M phase entry, with increases in G2/M phase populations at day 5 (27.0% vs. 6.20%). si*DSS1* also markedly induced the number of sub-G1 (dead) cells in drug-resistant MDA-MB-231 (24.8%, compared with 3.99% for siCtrl).

**Figure 5:**
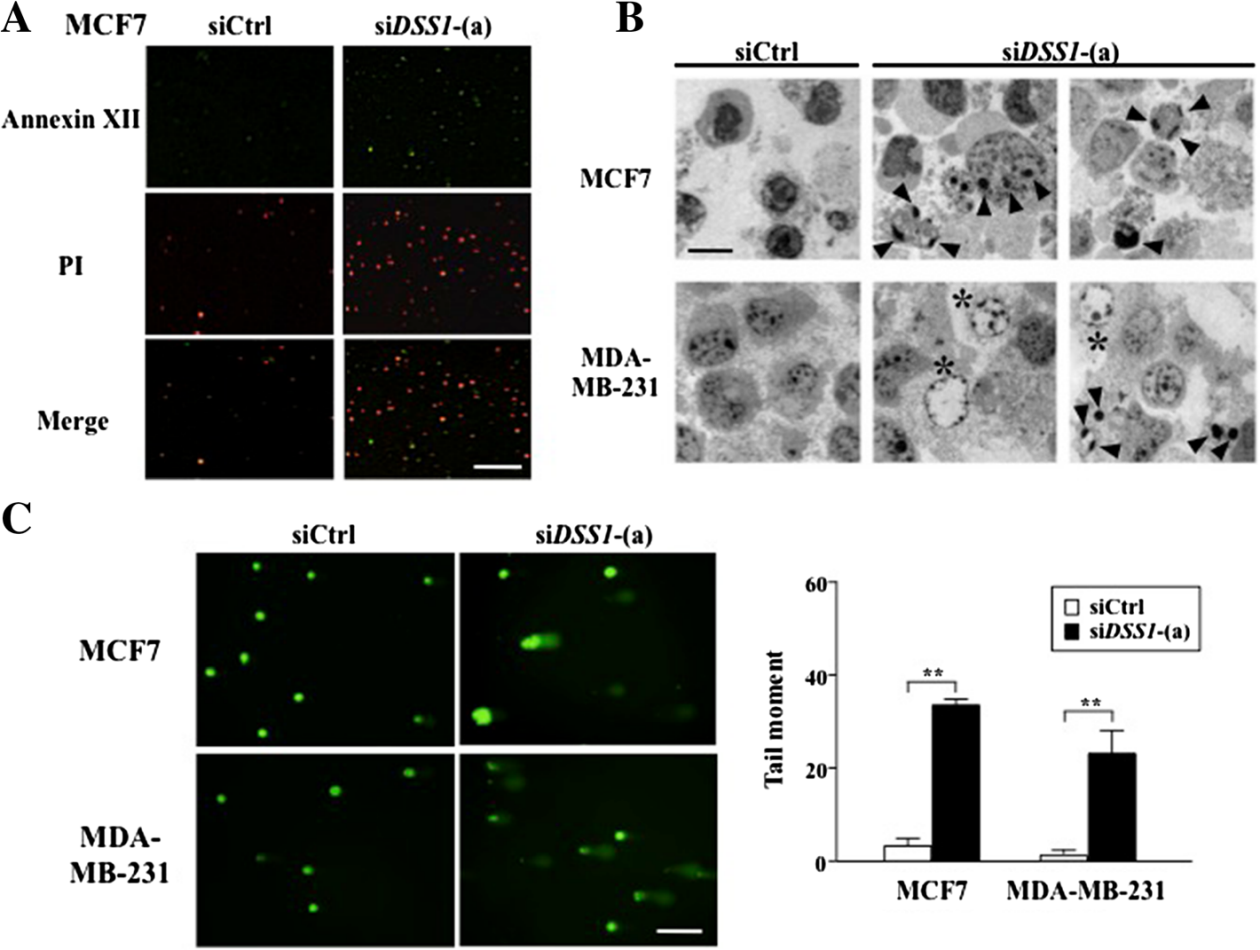
**Effect of *DSS1 *knockdown on apoptotic cell death in breast cancer cells. (A)** To examine the stage of cell death, live cells were stained with Annexin XII, which identifies the early apoptotic stage, in combination with PI staining; cells were marked as Annexin XII^+^PI^-^ (green; early apoptotic), Annexin XII^+^PI^+^ (doubly merged yellow; the intermediate stage), or Annexin XII^-^PI^+^ (red; late apoptotic/necrotic). Scale bar indicates 100 μm. **(B)** EM analysis was carried out at day 5 after treatment with siCtrl or si*DSS1*-(a). Arrowheads show the chromatin condensation typical of cell death, and asterisks indicate cell death with atypical chromatin accumulation and density. Scale bar indicates 500 μm. **(C)** An alkaline comet assay was carried out to detect the DNA damage resulting from both single-strand and double-strand breaks and evaluated as in Figure 3. Statistical significance was assessed by Student’s t-test, with **P < 0.01. Scale bar indicates 200 μm.

Treatment of MCF7 cells with si*DSS1* caused apoptosis, as detected by staining with Annexin XII and propidium iodide (PI). Representative Annexin XII^+^PI^-^ (early apoptotic) and Annexin XII^+^PI^+^ (late apoptotic/necrotic) cells are shown in Figure 5A. We further examined cell morphology at day 5 after si*DSS1* treatment using electron microscopy (EM). si*DSS1* treatment induced cell death in MCF7 cells, along with typical apoptotic changes including chromatin condensation, enlarged nuclei and smaller nuclei compared to the siCtrl-treated cells (Figure 5B; chromatin condensation marked with arrowheads). si*DSS1* treatment also caused apoptotic cell death in MDA-MB-231, with condensed chromatin and smaller nuclei relative to the control cells (Figure 5B; marked with arrowheads); however, some of these cells also showed atypical cell death morphologies, with enlarged cell size accompanied by electron microscopically void nuclei containing finely condensed chromatin-like structures adjacent to the inner nuclear membrane (Figure 5B; marked with asterisks). Because MDA-MB-231 do not seem to arrest at M phase entry, presumably due to p53 mutation, the atypical cell death observed in si*DSS1*-treated MDA-MB-231 might represent cell death occurring at various cell cycle stages. Finally, while the cell morphologies were not identical, si*DSS1*-treated MCF7 and MDA-MB-231 cells showed similar extents of DNA damage in the alkaline comet assay (Figure 5C).

**Figure 6:**
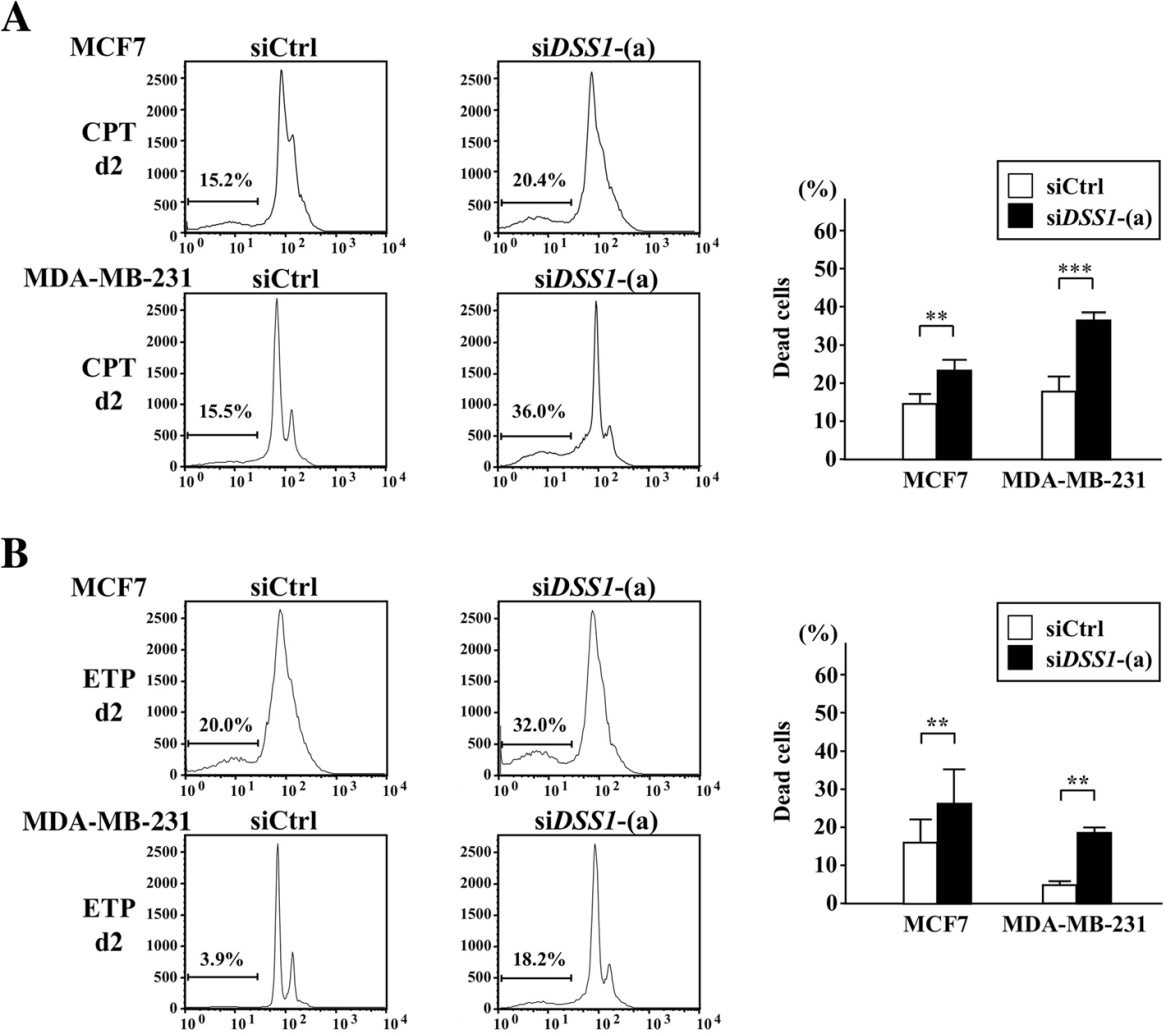
**Effect of *DSS1 *knockdown on the drug sensitivity of MCF7 and MDA-MB-231 cells.** Drug sensitivity was examined in *DSS1* knockdown cells with an optimized dose of CPT (50 μM) and ETP (50 μM). MCF7 and MDA-MB-231 cells were first treated with si*DSS1* for 2 days and then cultured with CPT **(A)** or ETP **(B)** for 2 days. **(A)** and **(B)** The number of cells in the sub-G1 (dead) cell populations was compared between si*DSS1*-(a)- or siCtrl-treated cells. The results of cell cycle profiling (left) were converted into the graph, which represents more than three independent experiments (right). Statistical significance was assessed by Student’s t-test, with **P < 0.01 and ***P < 0.001.

To confirm the effect of DSS1 on breast cancer cells, we investigated the effect of *DSS1* knockdown on cell cycle and cell death in breast cancer cells during early stages of treatment with CPT or ETP. *DSS1* knockdown markedly increased the number of cells in the sub-G1 apoptotic population in both MCF7 and MDA-MB-231 cells treated with CPT as early as day 2 (Figure 6A). Similarly, *DSS1* knockdown increased the sub-G1 population in both breast cancer cell lines treated with ETP (Figure 6B). Because MDA-MB-231 cells, which are of mesenchymal origin and have a triple-negative phenotype [22], are well known to be resistant to several DNA-damaging agents, these data suggested that *DSS1* depletion can increase chemosensitivity in drug-resistant breast cancer cells with either wild type or mutant p53.

## Discussion

Here, we demonstrate for the first time that DSS1 over-expression can be used as an early diagnostic marker for poor prognosis in cases of breast cancer. Because DSS1 maintains BRCA2 expression by regulating its ubiquitin-dependent degradation [23] and because the BRCA1:BRCA2 imbalance promotes tumorigenesis by increasing genomic instability [24], we predicted that increased expression of DSS1 could suppress breast cancer development. However, unexpected results have been observed in breast cancer cases. BRCA2 over-expression, rather than decreased expression, is associated with a poor survival rate [13] and poorer histological findings [11] in breast cancer cohorts. Our finding agrees with previous observations in cervical cancers with high DSS1 expression [25] and in breast cancers with high BRCA2 expression [11,13,14]. In a cervical cancer cohort [25], human papilloma virus oncoprotein E6 was found to bind to p53 and targets it for degradation through the ubiquitin-proteasome system, which also regulates BRCA2 stabilization. The expression of DSS1 is upregulated in cancerous regions compared to normal ones. The high DSS1 expression that potentially maintains high-level BRCA2 expression might cause or enhance breast cancer proliferation and/or drug resistance.

Cells with insufficient levels of *BRCA* genes are unable to repair DNA damage during the cell cycle and will eventually die. Tumors with germline BRCA mutations accompany the capacity to escape cell cycle checkpoints, which is in accordance with the low incidence of mutant BRCA-mediated sporadic tumorigenesis. BRCA2 interacts with RAD51 and BRCA1 to mediate DNA repair, P/CAF to mediate histone acetylation, BRAF-35 to mediate cell cycle regulation, and DSS1 [26]. DNA double-stranded breaks (DSBs) cause severe DNA damage, leading to replication arrest. BRCA1 and BRCA2 are involved in the repair of DSBs through their interaction with RAD51. BRCA2 is primarily involved in HR repair, but BRCA1 is involved in alternative DNA repair by non-homologous end joining (NHEJ) [27]. These DNA repair pathways are important for maintaining the integrity of the genome against the DSBs that occur at various phases of the cell cycle [28]. HR is principally involved in an error-free DNA repair mechanism that protects the genome during the S phase of cell cycle when the allelic genes on chromosomes are in close proximity [29]. Transcription-coupled DNA damage occurring at the other phases of the cell cycle is repaired by the NHEJ, which is required for prompt DNA repair in rapidly proliferating cells regardless of small errors in the genome [30]. The cellular genome is presumably maintained by the balanced regulation of these two DNA repair mechanisms [31].

The human DSS1 is homologous to Sem1, a component of the *Saccharomyces* TREX2 complex, which is composed of Sac3/Thp1/Sem1 in *Saccharomyces cerevisiae.* Thus, it is likely to be a functional component of the GANP/PCID2/ENY2/centrin/DSS1 complex involved in mRNA export in mammalian cells [32]. Many studies have addressed the character and function of the individual components of TREX2 complex in mammals [33]. Loss of any component of the mammalian TREX2 complex elicited a defect either in mRNA export or in the regulation of cell cycle progression, implying that each TREX2 component has an important individual function. Most of the defects associated with loss of the TREX2 component involve either replication or survival. In particular, DSS1 plays a role in stabilization of BRCA2 through regulation of its ubiquitin-dependent proteolytic degradation [23]. This may suggest a unique function for DSS1 in the organization of the ribonucleoprotein complex during various processes: transcription, nuclear to cytoplasmic export, and translation.

The p53^high^/*DSS1*^high^ group showed a worse prognosis in comparison with the p53^low^/*DSS1*^high^ group in breast cancer cases (Figure 1B), suggesting that the increased p53 status accelerates the effect of DSS1 over-expression on tumor progression under regular clinical treatments. This effect was not simply represented in the CPT-treatment of tumor cells between p53-wild type MCF7^DSS1^ and p53-mutated MDA-MB-231^DSS1^ (Figure 2C), while both of which sustained similar DNA damages by CPT under DSS1 over-expression (Figure 3). si*DSS1*, however, rendered both tumor cells very sensitive to CPT and ETP (Figure 6), suggesting that the effect of si*DSS1* appears dominantly on chemosensitivity of breast cancer cells irrespective with *TP53* status in the short-term culture. DSS1 might be involved not only in the proteolytic degradation of p53 [20], the stabilization of BRCA2 [23] and the process of DNA repair but also in the cell survival against anti-cancer drugs.

Regarding the cause of the poor prognosis in high DSS1-expressing breast cancers, an intriguing observation is the marked enhancement of drug sensitivity in highly drug-resistant MDA-MB-231 breast cancer cells by *DSS1* knockdown (Figure 4). Drug resistance is caused by various biological mechanisms in cancer cells, including pharmacokinetic resistance and/or cellular resistance. Beside the cellular drug transporter systems that allow multi-drug resistance of breast cancer cells, decreases in various nuclear enzymes could also cause multi-drug resistance. Decreased activity of topoisomerases has been described in several drug-resistant cancer cells including breast cancers [34]. Defects in cellular signaling pathways leading to apoptosis and DNA repair could result in multi-drug resistance, as can p53 insufficiency, decreases in ceramide levels [35], DNA alkyl-transferase activation [36], and problems with the mismatch DNA repair system [37,38]. Decreased expression of BRCA2 has been suggested to be a marker for a favorable response to docetaxel in breast cancer [39]. *BRCA2* knockdown has been proposed to as a means to synergistically potentiate cisplatin and melphalan treatment [40]. High DSS1 expression, which potentially stabilizes BRCA2 and maintains cancer cell proliferation, could increase drug resistance under the standard clinical regimens of breast cancer treatment, presumably resulting in decreased disease-free survival.

## Conclusions

Sporadic cases of breast cancer with high expression of DSS1 showed a worse prognosis with respect to RFS; however, increased DSS1 expression was not correlated with increased proliferation or tumor grade. DSS1 over-expression increased the resistance of breast cancer cells to DNA-damaging drugs; conversely, *DSS1* knockdown rendered breast cancer cells more sensitive to these drugs. These results clearly indicate that *DSS1* knockdown in combination with chemotherapy might be effective for treatment of breast cancer.

## Abbreviations

HR: Homologous recombination; RT-PCR: Reverse transcription-PCR; qRT-PCR: Quantitative RT-PCR; DSS1: Deleted in split hand/split foot 1; TREX2: Transcription/exportation 2; RFS: Relapse-free survival; BCSS: Breast cancer specific survival; ERα: Estrogen receptor α; PgR: Progesterone receptor; CPT: Camptothecin; ETP: Etoposide; NHEJ: Non-homologous end joining; siRNA: Small interfering RNA.

## Competing interests

The authors declare that they have no competing interests.

## Authors’ contributions

AR, KK, MY-I, MK, PM, SP, TS, and ST acquired data and performed the statistical analysis. MY-I, YY, and HI performed the cohort analysis. KK designed the study, analyzed and interpreted the data, and drafted the manuscript. NS designed the study, analyzed and interpreted the data, and drafted and approved the manuscript. All authors read and approved the final manuscript.

## Pre-publication history

The pre-publication history for this paper can be accessed here:

http://www.biomedcentral.com/1471-2407/13/562/prepub

## Supplementary Material

Additional file 1: Figure S1 Differences between the *DSS1*^high^ and the *DSS1*^low^ groups based on qRT-PCR. Patients having tumors with high DSS1 expression were classified by the mRNA level as the *DSS1*^high^ group (*DSS1*/*β-actin* ratio > 136). Boxes represent the mean and 70% confidence intervals; *bars,* standard deviations. Click here for file

Additional file 2: Figure S2 Establishment of DSS1 over-expressed MCF7 and MDA-MB-231 cells. **(A)** Schematic diagram of retroviral vectors (pFB-IRES-GFP and pFB-DSS1-IRES-GFP). **(B)** Increased expression of *DSS1* transcripts in DSS1 over-expressed MCF7 and MDA-MB-231 cells. Representative data is shown from three independent experiments. Click here for file

Additional file 3: Figure S3 Drug sensitivity in DSS1 over-expressed MCF7 and MDA-MB-231 cells. **(A)** and **(B)** The effect of DSS1 over-expression on drug sensitivity was examined in MCF7 and MDA-MB-231 cells at day 2 and day 3 after treatment with ETP (50 μM). n.s.: not significant. Click here for file

Additional file 4: Figure S4 Effect of si*DSS1* on DSS1 expression and cell proliferation. **(A)** si*DSS1*s on two independent sequences were transfected into MCF7 and MDA-MB-231 cells. The expression levels of *DSS1* transcripts were measured by qRT-PCR. Similar knockdown efficiency was observed in both si*DSS1*-(a) and si*DSS1*-(b) transfected cells. The data are representative of three independent experiments. **(B)** The expression levels of DSS1 were measured by Western blot in siCtrl-, si*DSS1*-(a)-, and si*DSS1*-(b)-treated cells. β-actin was used as a loading control. **(C)** Effect of si*DSS1*-(b) was similar in cell proliferation (MTT assay) compared with that of si*DSS1*-(a) shown in Figure 4. Statistical significance is shown by the Student’s t-test calculation with **P < 0.01 and ***P < 0.001. Click here for file

Additional file 5 Western blot analysis. Click here for file
