# Supplementary figures and images for: Breast cancers with high DSS1 expression that potentially maintains BRCA2 stability have poor prognosis in the relapse-free survival
Source: BMC Cancer. 2013 Dec 1;13:562. doi: 10.1186/1471-2407-13-562 (PMC4219476; doi:10.1186/1471-2407-13-562)

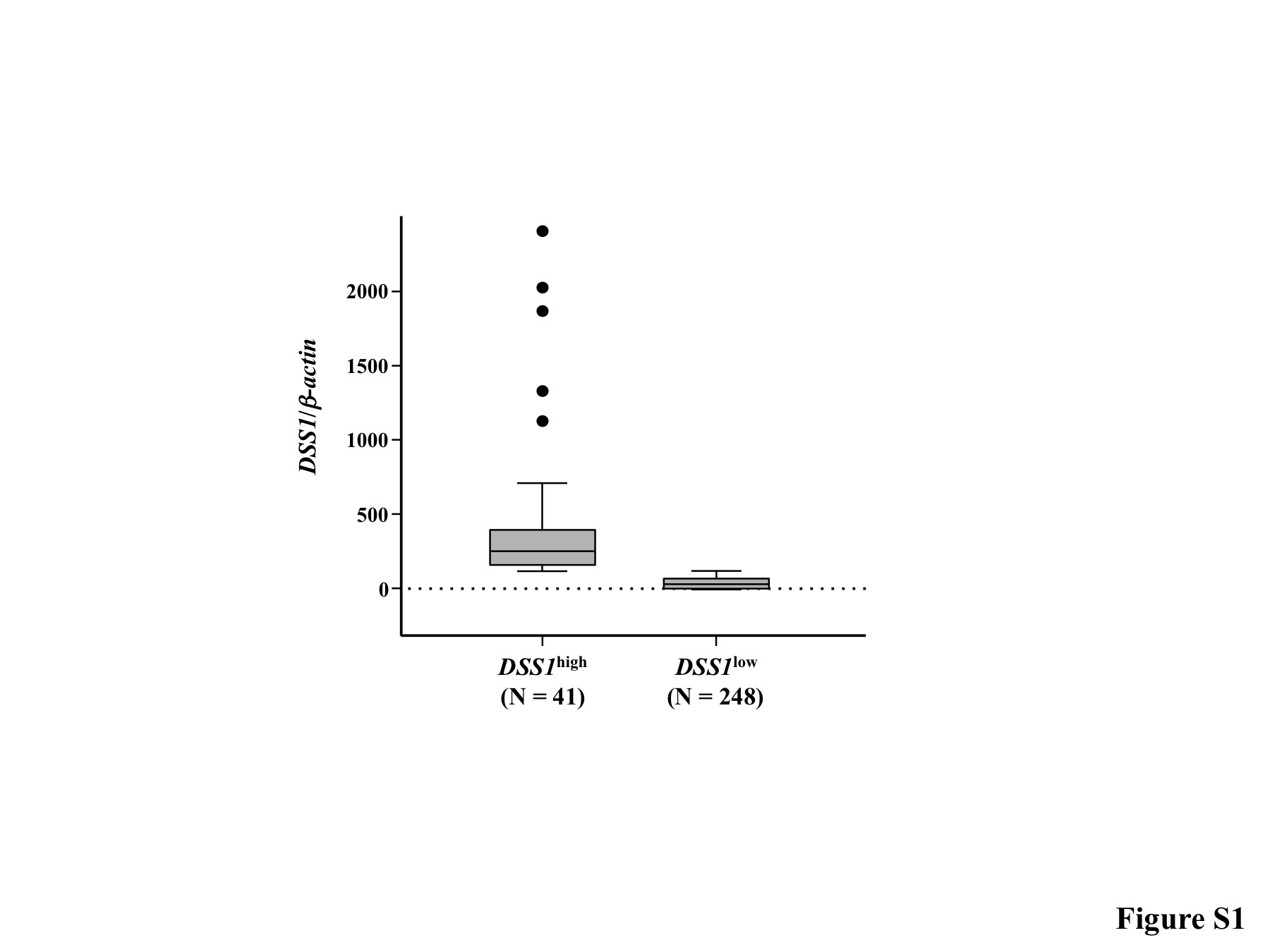

Supplement: Additional file 1: Figure S1 — Differences between the DSS1high and the DSS1low groups based on qRT-PCR. Patients having tumors with high DSS1 expression were classified by the mRNA level as the DSS1high group (DSS1/β-actin ratio > 136). Boxes represent the mean and 70% confidence intervals; bars, standard deviations. [file 1471-2407-13-562-S1.jpeg]

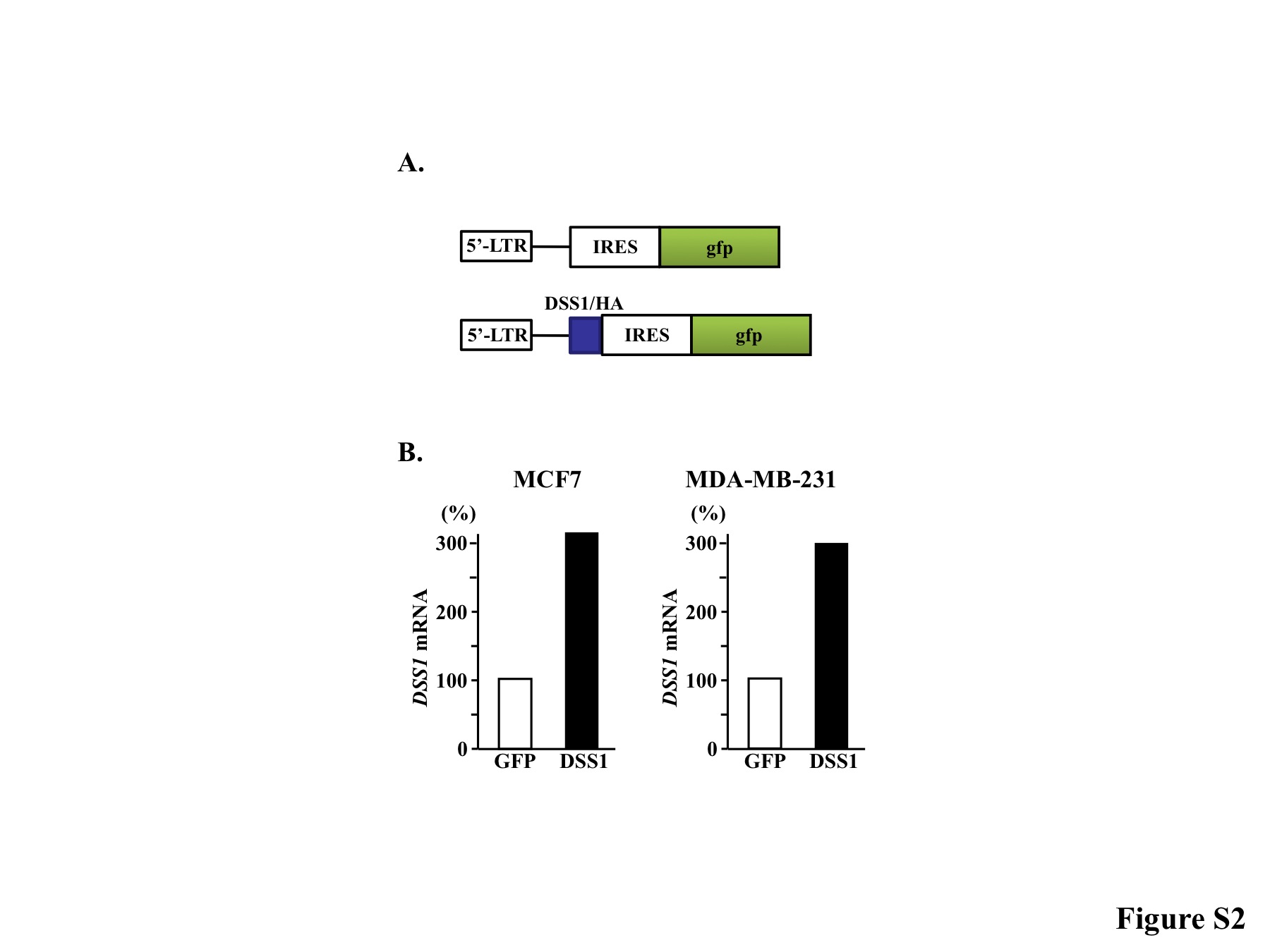

Supplement: Additional file 2: Figure S2 — Establishment of DSS1 over-expressed MCF7 and MDA-MB-231 cells. (A) Schematic diagram of retroviral vectors (pFB-IRES-GFP and pFB-DSS1-IRES-GFP). (B) Increased expression of DSS1 transcripts in DSS1 over-expressed MCF7 and MDA-MB-231 cells. Representative data is shown from three independent experiments. [file 1471-2407-13-562-S2.jpeg]

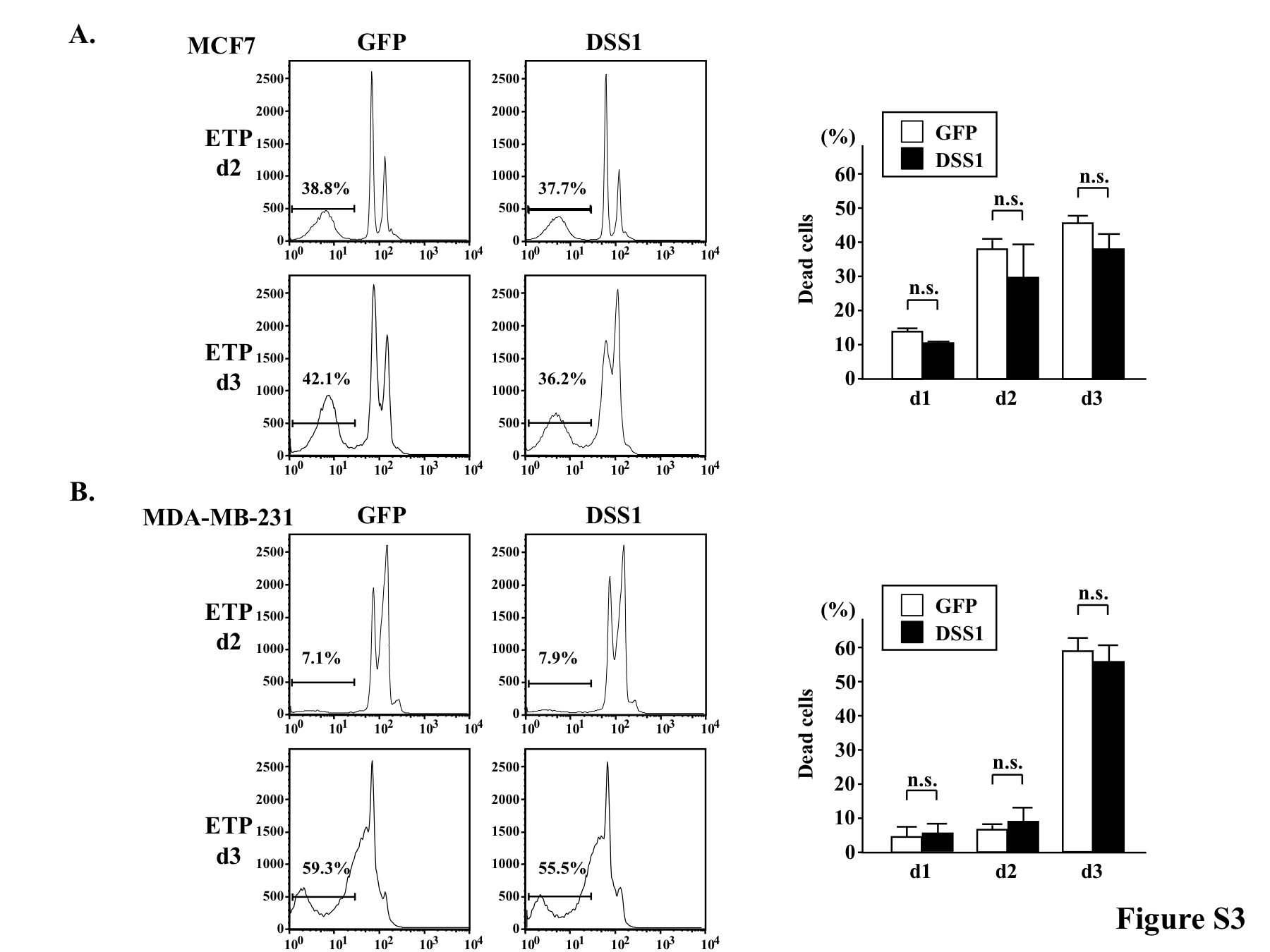

Supplement: Additional file 3: Figure S3 — Drug sensitivity in DSS1 over-expressed MCF7 and MDA-MB-231 cells. (A) and (B) The effect of DSS1 over-expression on drug sensitivity was examined in MCF7 and MDA-MB-231 cells at day 2 and day 3 after treatment with ETP (50 μM). n.s.: not significant. [file 1471-2407-13-562-S3.jpeg]

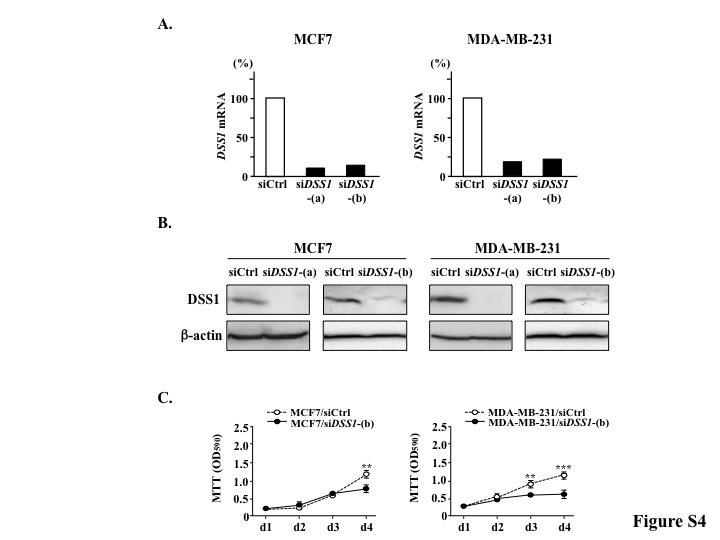

Supplement: Additional file 4: Figure S4 — Effect of siDSS1 on DSS1 expression and cell proliferation. (A) siDSS1s on two independent sequences were transfected into MCF7 and MDA-MB-231 cells. The expression levels of DSS1 transcripts were measured by qRT-PCR. Similar knockdown efficiency was observed in both siDSS1-(a) and siDSS1-(b) transfected cells. The data are representative of three independent experiments. (B) The expression levels of DSS1 were measured by Western blot in siCtrl-, siDSS1-(a)-, and siDSS1-(b)-treated cells. β-actin was used as a loading control. (C) Effect of siDSS1-(b) was similar in cell proliferation (MTT assay) compared with that of siDSS1-(a) shown in Figure 4. Statistical significance is shown by the Student’s t-test calculation with **P < 0.01 and ***P < 0.001. [file 1471-2407-13-562-S4.jpeg]
