# Supplementary material for: Breast cancers with high DSS1 expression that potentially maintains BRCA2 stability have poor prognosis in the relapse-free survival
Source: BMC Cancer. 2013 Dec 1;13:562. doi: 10.1186/1471-2407-13-562 (PMC4219476; doi:10.1186/1471-2407-13-562)
Supplement: Additional file 5 — Western blot analysis. [file 1471-2407-13-562-S5.docx]

**Additional File**

**Supplementary Methods**

**Western blot analysis**

Cells treated with siDSS1-(a) or -(b) were collected and lysed with RIPA buffer (50 mM Hepes pH 7.5, 150 mM NaCl, 1 mM EDTA, 1% Nonidet P-40, and protease inhibitor cocktail). Equal amount of protein from the lysate was separated on a SDS-PAGE (18%) gel, then transferred onto PVDF membranes (GE Healthcare, Piscataway, NJ) prewetted in 100% methanol for 10 sec using 25 mM potassium phosphate buffer [23]. The blot was probed with the Ab against DSS1 (Proteintech, Chicago, IL) in combination with horseradish peroxidase-conjugated anti-rabbit Ab. Anti-β-actin Ab (Sigma-Aldrich) was used as an internal control.

**Supplementary Figure legends**

**Figure S1. Differences between the *DSS1*^high^ and the *DSS1*^low^ groups based on qRT-PCR.** Patients having tumors with high DSS1 expression were classified by the mRNA level as the *DSS1*^high^ group (*DSS1*/*β-actin* ratio > 136). Boxes represent the mean and 70% confidence intervals; bars*,* standard deviations.

**Figure S2. Establishment of DSS1 over-expressed MCF7 and MDA-MB-231 cells.** (**A**) Schematic diagram of retroviral vectors (pFB-IRES-GFP and pFB-DSS1-IRES-GFP). (**B**) Increased expression of *DSS1* transcripts in DSS1 over-expressed MCF7 and MDA-MB-231 cells. Representative data is shown from three independent experiments.

**Figure S3. Drug sensitivity in DSS1 over-expressed MCF7 and MDA-MB-231 cells.** (**A**) and (**B**) The effect of DSS1 over-expression on drug sensitivity was examined in MCF7 and MDA-MB-231 cells at day 2 and day 3 after treatment with ETP (50 μM). n.s.: not significant.

**Figure S4. Effect of si*DSS1* on DSS1 expression and cell proliferation.** (**A**) si*DSS1*s on two independent sequences were transfected into MCF7 and MDA-MB-231 cells. The expression levels of *DSS1* transcripts were measured by qRT-PCR. Similar knockdown efficiency was observed in both si*DSS1*-(a) and si*DSS1*-(b) transfected cells. The data are representative of three independent experiments. (**B**) The expression levels of DSS1 were measured by Western blot in siCtrl-, si*DSS1*-(a)-, and si*DSS1*-(b)-treated cells. β-actin was used as a loading control. (**C**) Effect of si*DSS1*-(b) was similar in cell proliferation (MTT assay) compared with that of si*DSS1*-(a) shown in Figure 4. Statistical significance is shown by the Student’s t-test calculation with ***P* < 0.01 and ****P* < 0.001.
